# Observations on the Polish Carbuncle

**Published:** 1829-07-01

**Authors:** 


### XXVIII.

### OBSERVATIONS ON THE POLISH CARBUNCLE AND AN EPIZOOTIC DISEASE AS ITS CAUSE. By A. T. HELBICH, M. D.

The pamphlet containing these observations has not come to our hands; and for the following short notice of it we are indebted to our transatlantic cotemporary, the AMERICAN JOURNAL of the MEDICAL SCIENCES, No. IV. for August 1828.

"The Polish carbuncle, which was thus denominated by Schmaltz, on account of the frequency of its occurrence in Poland, is the same disease which has been indicated by Chaussier, and other French writers by the name of *pustule maligne ou gangreneuse, bouton malin, puce maligne, &c.* and by the Germans as *brandblatter, schwartzeblatter, &c.* There can be little doubt but that this terrible disease has occasionally existed from the remotest antiquity, and in various countries under similar circumstances, though our author believes it to have been confounded with the gangrenous anthrax of medical writers. Ramazzini relates that it raged in Italy in 1609; Schroek in 1712, and Buchner in 1726, speak of it as existing in Germany and Poland; Chaignebrun describes it at Paris in 1757, and Hartmann observed it the following year in Finland; Berlin describes this disease as having destroyed two hundred negroes at Guadaloupe in 1774. Since that period various writers have treated of this disease, but few of them have enjoyed opportunities of observation equal to Dr. Helbich, whose account is given with that vigorous raciness, so peculiarly distinctive of originality.

"As the Polish carbuncle, or gangrenous pustule is communicated to the human race by simple contact from diseased domestic animals, Dr. Helbich properly commences with a sketch of the predisposing causes and form of the disease which affects these creatures. He states that when the spring comes on rapidly and pleasantly, the sudden melting of the ice and snow swells the water-courses, which overflow the whole soil of low regions. As the summer approaches the waters subside, leaving numerous pools, that soon exhale fetid vapours, and afford an unwholesome drink to the cattle, which are all day exposed to an ardent sun. The inundation spreads a filthy slime over vegetation, which is subsequently filled with putrescent insects. Cattle using such food, soon begin to grow unhealthy, prostration of strength ensues, and a disposition to putrid and destructive fevers supervenes.

"Three stages may be observed in this disease among domestic animals. The inceptive, the period of increase, and that of termination. In the first the animal loses appetite, becomes languid, weak, somewhat thinner, the eyes lose their lustre, and sometimes have a subinflammatory redness. Cows cease to yield milk. After various intervals chills ensue, which are even more intense when the attack comes on in Summer. The sadness and feebleness of the animal increasing, fever begins to rise, the bowels are constipated, and the animal is wrung by a painful tenesmus. Ruminant animals cease to ruminate. The left side becomes tumid, and painful to the touch. Sometimes the attack presents differences according as the appetite for food remains or is exceedingly increased, or scarcely any fever comes on. Occasionally the disease comes on with few obvious symptoms.

"The fever persisting in severity, is followed by the greatest prostration of strength, and the increase of the disease is indicated by the tottering of the hinder limbs. Soon after the animal falls down, having the neck very much stretched out, or with the mouth drawn to the left side. The teeth are gnashed. The whole body convulsed, and the internal sufferings extort deep groans. The bowels are totally obstructed. Blood burned to blackness, flows most copiously during the tenesmus, or in consequence of the internal convulsions from the prolapsed rectum. Sometimes I have observed an animal to fall down and expire suddenly, without exhibiting any of the more violent symptoms.

"The termination of the disease is twofold; sometimes the worst symptoms are protracted for some days, and the fever diminishes; fetid evacuations, at first copious, become at length natural; rumination returns, and, after a long convalescence, the animal recovers its health. But, more frequently, the disease attains its highest degree. The skin is studded with knots or gangrenous carbuncles; chronic spasms end in universal tetanus; a viscid saliva flows from the gaping mouth, the respiration is only continued by painful convulsive efforts, and after long agonies the poor beast expires.

"On dissection, the lungs are found loaded with black blood, spoiled, and sometimes so soft that they break with the least touch. The liver is sometimes natural in appearance, at other times beset with squalid ulcers; the gall-bladder is copiously filled with black bile; the intestinal tube is inflamed and filled at different parts with a colluvies of black and fetid blood. Most frequently, however, the characteristic sign of the disease is found in the spleen, as this viscus is of thrice the natural size, rotten, and as if composed of nothing but clotted blood, showing no evidences of its proper structure, and bursting immediately when dropped on the ground.

"Although this fever attacks all domestic animals, it is not contagious, as from whatever cause it may arise it affects one herd without extending to another. It is often observed that healthy calves suck diseased cows without taking the disease; and at times of a herd confined to the same pasture, part will perish of the malady without the other part being at all affected.

"During the ten years which Dr. Helbich practised in Poland, he treated two hundred patients labouring under the carbuncle, and never saw a single instance in which there was any reason to believe in the spontaneous origin of the disease in the human subject, but that, 1st. Whenever the putrid fever raged among the cattle, men were affected with the Polish carbuncle. 2d. The disease was most especially frequent wherever the epizootic raged most violently. 3d. Those persons only took the contagion, who were by their occupations obliged to be about cattle, as farmers, herdsmen, butchers, tanners, &c. 4th. The Polish carbuncle only seized upon the naked parts of the body fitted to receive the poison, as the face, arms, and feet; rarely were other parts affected. 5th. Another argument in favour of the communication of the disease from without, is that it first appeared externally, and afterwards the internal symptoms came on.

"The disease was communicated by simple contact of the blood of the diseased living animal, or any of the excretions, or by merely touching the skin of the nostrils, as has been proved by many authorities. I shall hereafter relate such an instance which fell under my own observation.

"The disease was also communicated by the contact of any part of an animal dead of the putrid fever. As all the solids and fluids of the animal contain the poison, it is not surprising that this mode of communication should be the most frequent. The country people, ignorant of the nature of the disease, killed the sickly cattle, either for the sake of their flesh, or for the purpose of diminishing their loss by the sale of their skins. Under any of these circumstances, naked parts of the human body were brought into immediate contact with the carcass, which poisoned with virulence, whether the integuments were sound or excoriated. Whether one part of the carcass were more poisonous than the other is uncertain, and not easy to be ascertained by experiment. The viscera, as if containing the seeds of the disease, appeared to be more noxious than other parts. A warm carcass infected more readily than a cold one. All parts of the surface of the human body take the infection with equal readiness; wherever the skin is touched by the poisonous fluids the deleterious principle is absorbed, and the gangrenous inflammation supervenes. The following instance is the one above referred to:—

"In the province of Kramsk, about the commencement of June, 1826, some cattle died very suddenly, when no person was known to have the Polish carbuncle, nor had the people observed any remarkable cause for the disease among the cattle. Three young rustics were engaged in stripping the hide from a dead ox, when a very robust and healthy country wench, about thirty years of age, passing by, fell to deriding the young men for skinning the dead animal. Seeing that she had offended them very much, she took to flight, and they chased her, caught and threw her on the ground, and with their bloody hands, as a good joke, smeared her face about the lips, her bosom, and neck. In three days afterwards, all the parts thus touched were affected with a violent itching, and broke out into carbuncles. From that time, many of the inhabitants of the province were affected by the disease, and an individual perished from neglect of all treatment. When the prefect heard of the dreadful state of the disease he requested me to go thither, and I found fourteen persons suffering from the carbuncle, and one dead, as just mentioned. The unfortunate woman above-mentioned was in the agonies of death when I arrived, and died a few hours afterwards, on the eighth day from the application of the matter, and on the fifth from the eruption.

"It is very singular that those who are engaged in skinning or cutting up the animals, which are uniformly killed by the Polish peasantry, as soon as they perceive them to be affected with the fever, are more liable to be attacked by the carbuncle than those who eat of the flesh. Dr. Helbich has seen as many as two hundred boors eat of such flesh, boiled and roasted, without the slightest injury, while the skinners invariably fell under the disease. A couple of rascally butchers, at the beginning of one of these epizootics, brought by stealth a diseased bullock into the town of Konin, which they slaughtered at night and exposed the next morning in the market for sale. Many of the inhabitants, and Dr. Helbich himself, purchased and ate of this beef, without the least injury, and the cheat might never have been discovered had not both the rogues in a few days afterwards been rewarded for their exploit by an attack of the dreaded carbuncle. Nevertheless it is by no means to be understood that disease may not be communicated by such food, which should be most carefully avoided.

"Dr. Helbich does not believe that the disease is communicable from one human body to another, without actual contact of the diseased part.

"The disease begins in the following manner: a few days after the communication of the infection, a slight colourless swelling, like a flea bite, is perceived, which is unaccompanied by increased heat. In a short time, and generally at the same moment, a pustule appears in the centre of the little prominence, never exceeding the size of a millet seed, and when examined by the microscope is seen to be filled with a viscid fluid. This pustule is productive of dreadful itching, and an irresistible propensity to scratch. When the epidermis is removed the pustule gradually increases, and is filled with an ichorous fluid, which at length becomes black. When the pustule is accidentally broken or opened with an instrument, a few drops of bloody humour escape, and the bottom of the little ulcer appears of a livid black, somewhat higher towards the edges, depressed in the centre, concave and scooped out like an umbilicus and secreting an ichor from all its surface. This gangrenous affection of the skin having advanced to the cellular texture, is at this time about the size of half a hazlenut. The disease soon after becomes more extensive, inflammation ensues in the neighbouring parts, gangrenous phlyctenæ form in the vicinity of the tumours, violent burning pains are felt, the skin becomes puffy and elastic; all the evidences of vast irritation show themselves; fever, nausea, vomiting, and all the signs of the participation of the stomach and bowels ensue, and after a struggle varying in duration, the unfortunate patient expires.

"We regret that it is not in our power to follow our author throughout his account, as we cannot otherwise do him the justice we wish. Such an extension would be incompatible with the design of these notices, and we must hasten to a conclusion.

"The disease is always to be arrested in the first stage, as it is entirely local. Excision with the knife or destruction by caustic potash are uniformly successful when properly employed. Dr. Helbich, for reasons which will present themselves to every reflecting mind, gives a general preference to the caustic, and he has been very successful in treating the disease. The other treatment consists principally of the administration of such materials as are suited to keep up the strength of the patient, and diminish excessive irritation. The inflammatory stage is so short as scarcely to allow of antiphlogistic measures. All local emollient applications are positively injurious and increase the gangrenous disposition. The dressings must be stimulating, nnd the country people frequently sprinkle the ulcers with salt, powder of cantharides, and other acrid and stimulating substances with advantage."

The foregoing document may give rise to interesting reflexions, not only as to the cause of the disease in animals, but as to the communication of it to the human race.

